# Propyl Gallate Plays a Nephroprotective Role in Early Stage of Diabetic Nephropathy Associated with Suppression of Glomerular Endothelial Cell Proliferation and Angiogenesis

**DOI:** 10.1155/2012/209567

**Published:** 2012-09-04

**Authors:** Shaojiang Tian, Junming Tang, Huihui Liu, Liping Wang, Jianming Shen, Junfeng Li, Yanjie Gan

**Affiliations:** ^1^Department of Nephrology, Renmin Hospital, Hubei University of Medicine, Hubei, Shiyan 442000, China; ^2^Clinic Medicine Research Institute, Renmin Hospital, Hubei University of Medicine, Hubei, Shiyan 442000, China; ^3^Department of Cardiology, Renmin Hospital, Hubei University of Medicine, Hubei, Shiyan 442000, China

## Abstract

There is growing evidence suggesting that glomerular endothelial cell proliferation and angiogenesis may be responsible for the pathophysiological events in the early stage of diabetic nephropathy. This study was designed to investigate the factors related to glomerular endothelial cell proliferation and glomerular angiogenesis and assess the effect of propyl gallate on preventing these disorders in diabetic rats. We found that glomerular hypertrophy, glomerular mesangial matrix expansion, and albuminuria were significantly increased in DN rats. CD31+ endothelial cells significantly increased in glomerulus of diabetic rats. Double immunofluorescence staining showed some structurally defective vasculus tubes in glomerulus. Real-time PCR and western blot demonstrated the glomerular eNOS expression remained at the same level, while remarkable decreased NO productions and suppressed eNOS activities were observed in diabetic rats. Treatment with propyl gallate improved glomerular pathological changes, reduced endothelial cell proliferation, decreased albuminuria, and restored eNOS activity, but did not alter eNOS expression. These data suggest that endothelial cell proliferation and immature angiogenesis may be the contributors to progression of DN. Propyl gallate is a potential novel therapeutic agent on prevention of diabetic nephropathy.

## 1. Introduction

Diabetic nephropathy is the most frequent cause of chronic kidney disease and usually quickly progresses to end-stage renal disease requiring renal replacement therapy. Early alterations in diabetic nephropathy include development of glomerular hyperfiltration and glomerular hypertrophy, followed by increased urinary albumin excretion and eventually leading to glomerular sclerosis [1]. Although intensified controls of hyperglycemia, hypertension, and proteinuria slow down the progression of DN, these therapies do not sufficiently retard the progression of DN [2].

The exact mechanism underlying the pathogenesis of DN is yet clear. However, there is ample evidence for the involvement of endothelial cell proliferation and angiogenesis, the development of new blood vessels from preexisting ones, in facilitating glomerular hypertrophy in early stage of DN [3]. In addition, some antiangiogenic agents ameliorate glomerular hypertrophy and hyperfiltration as well as urinary albumin excretion in early stage of DN [4, 5]. The results of these studies strongly suggest that glomerular angiogenesis may be responsible for the pathophysilogical events in the early stage of DN.

Microalbuminuria is considered an early feature of incipient DN [6]. However, albuminuria is more than just a marker; it is also believed to be a promoter of the progression of DN [7]. If glomerular angiogenesis does influence DN progression, the ameliorating or worsening of glomerular angiogenesis would provide more close association with the severity of albuminuria. But, whether and how glomerular angiogenesis implicated in albuminuria is yet to be established. It is believable that the integrity of filtration barrier is crucial for inhibiting protein exfiltration, and intensive endothelial proliferation and angiogenesis may result in structure damage of glomerular filtration barrier. In terms of podocyte is a kind of deproliferation cells [8], rampant endothelial cell proliferation and neovascularization may result in nude endothelial tubes. Investigating the structural characteristics of newly formed tube may provide us with a deeper understanding of implication of angiogenesis in initiation and progression of DN.

For mechanisms of glomerular endothelial cell proliferation and angiogenesis, previous studies suggested uncoupling of VEGF-NO axis play a key role in those abnormal events [3]. VEGF is a well known angiogenic fictor, while NO plays a critical role in the maintenance of endothelial cell differentiation state. However, once the negative regulation is lost due to reduced NO availability, VEGF action may be become excessive and lead to endothelial cell proliferation and angiogenesis [9]. Considering the high expression of VEGF is a common phenomenon in diabetic nephropathy [5], the factors related to NO deficiency are the most concerned for endothelial cell proliferation. Our recent studies have shown that oxidative stress may implicated to glomerular NO deficiency through inhibiting eNOS activity, and an antioxidant agent—propyl gallate—can restore eNOS activity [10]. However, whether propyl gallate can inhibit the proliferation of endothelial cells and arrest the progression of diabetic nephropathy is yet to be explored.

In the present study, we demonstrate that unlimited endothelial proliferation and tube formation may result in podocyte-missing glomerular vascula in early stage of DN. Those immature vessels may be the culprit of leaking filtration barrier and promoting factor of DN progression. The study suggests that reduced NO production in the glomeruli is involved in glomerular endothelial proliferation, and the NO insufficiency maybe mainly resulted from eNOS inactivation, but not from the decrement in eNOS expressions. Our data also show that propyl gallate inhibited glomerular endothelial cell proliferation and angiogenesis, and this antiangiogenesis effect may be through recovering eNOS activity.

## 2. Material and Methods

### 2.1. Induction of Diabetes and Treatment Protocols

 The experimental protocol was approved by the Animal Ethics Review Committee for Enamel Experimentation of Hubei University of Medicine. Male rats were fed a standard pellet laboratory diet and were provided with tap water. Type 1 diabetes was induced by an intraperitoneal injection of STZ dissolved in 10 mmol/L sodium citrate at 50 mg/Kg body wt. into 8-week-old rats after overnight fasting. Control rats received injections with buffer alone. Six days after the injections, rats with blood glucose in the range of 13.9–22.2 mmol/L were divided into three groups: (1) nondiabetic control group, (2) diabetic group, and (3) propyl gallate treatment diabetic group. Rats of group 3 received intraperitoneal injections of propyl gallate (5 mg/Kg body wt) six days a week. Blood glucose and urine samples were monitored every week, and when needed, diabetic rats were given supportive insulin treatment to maintain blood glucose in the range of 13.9–22.2 mmol/L and to prevent ketosis. No rats died, and no signs of apparent exhaustion were observed during the experimental period. Eight weeks after induction of diabetes mellitus, the rats were anesthetized with pentobarbital sodium (50 mg/kg body wt). The kidneys were perfused with ice-cold PBS (pH 7.4). The 1/2 right kidney from each rat was fixed in 4% paraformaldehyde, 1/4 embedded in paraffin for pathological analysis, and other 1/4 embedded in Tissue-Tek OCT for immunofluorescence. The renal cortex of another part of right kidney and the left kidney from each rat was cut into small pieces, and glomeruli were isolated by the mechanical graded sieving technique. After isolation, the purity of the final suspension was determined by light microscopic examination. On average, tubular contamination was <5%. The glomerular suspension was then used for NO-production assay, ROS activity analysis, protein isolation, and RNA isolation. The experimental protocol was approved in advance by the Ethics Review Committee for Animal Experimentation of the Hubei University of Medicine.

### 2.2. Urine Examination

 Eight weeks after the induction of diabetes, individual 24 h urine sample collections were performed using metabolic cages. Urinary creatinine levels were measured by the enzymatic colorimetric methods. Urinary albumin concentration was measured by nephelometry using anti-rat albumin antibody. Results of urinary albumin were normalized to urinary creatinine levels and expressed as urinary albumin-to-creatinine ratio. After urine samples collection, rats were anesthetized with pentobarbital (50 mg/Kg body wt, ip) and killed, and then kidneys were obtained.

### 2.3. Histological Analysis

 Kidney pieces fixed in paraformaldehyde were embedded in paraffin. Sections (4 *μ*m) were stained with periodic acid Schiff for light microscopic observation to determine glomerular hypertrophy and mesangial matrix extension.

### 2.4. Immunofluorescence

 Kidney pieces embedded in Tissue-Tek OCT compound were frozen gently in liquid nitrogen and cut on a cryostat microtome into 4 *μ*m sections that were affixed to microscope slides. For CD31 single immunoflurescence, sections were incubated overnight with affinity-purified mouse anti-rat CD31 antibody at a 1 : 100 dilution. Secondary antibody, an FITC-conjugated goat anti-mouse antibody was applied at a 1 : 1000 dilution, and immunofluorescence photomicrographs were obtained at a ×400 magnification for a 100 ms exposure time. For CD31 and podocalyxin double immunoflurescence, rabbit anti-rat podocalyxin antibody at a 1 : 100 dilution was applied at the same time as adding the CD31 antibody, and secondary antibody also included FITC-conjugated goat anti-mouse antibody and TRITC-conjugated goat-anti-rabbit antibody at a same dilution.

### 2.5. RNA Extraction and Quantitative Real-Time RT-PCR

Total RNA was extracted from isolated glomeruli using TRIzol (Invitrogen, USA) according to the manufacturer's instructions. Reverse transcription of 1 *μ*g RNA was performed using 100 pmol random OligA primers (Amersham Pharmacia Biotech) and 300U SuperScript RNAse H (GIBCO BRL) with the mixture of 5 *μ*L buffer, 10 *μ*mol dNTP, 0.1 mmol DTT, and 60U RNAsin (Boehringer Mannheim) at 42°C for 1 h. cDNA was diluted 1 : 5 with autoclaved deionized water, and 5 *μ*L of the diluted cDNA was added to Lightcycler-Mastermix, 0.5 *μ*mol/L specific primer, 3 mmol/L MgCI_2_, and 2 *μ*L Master SYBR green (Roche Diagnostics, Mannheim). This reaction mixture was filled up to a final volume of 20 *μ*L with water. PCR was carried out in a real-time PCR cycler (Lightcycler, RocheDiagnostics). The program was optimized and performed finally as denaturation at 95°C for ten min followed by 40 cycles of amplification (95°C for 10 s, 65°C for 10 s, and 72°C for 10 s). The temperature ramp rate was 20°C per s. At the end of each extension step, the fluorescence was measured to quantitate the PCR products. After completion of the PCR, the melting curve of the product was measured by temperature gradient from 65 to 95°C at 0.2°C per s with continuous fluorescence monitoring to produce a melting profile of the primers. The following oligonucleotide primers for eNOS were used: sense—CTG CTG CCC GAG ATA TCT TC and antisense—CAG GTA CTG CAG TCC CTC CT.

### 2.6. Western Immunoblotting

Portions of isolated glomerular samples were homogenized in lysis buffer, supplemented with protease inhibitors and sodium orthovanadate, and spun at 14,000 ×g to pellet the nuclei and large cellular fragments. The supernatant protein concentrations were measured by the Lowry assay (Bio-Rad). Appropriate volumes of the supernatant (100 *μ*g/lane) were mixed with an equal volume of sample buffer (100 mmol/L Tris·HCl, pH 6.8, 4% SDS, 20% glycerol, 10% 2-mercaptoethanol, and 0.02% bromophenol blue) and subjected to SDS-PAGE using 12.5% acrylamide gels. The proteins were transferred by semidry electroblotting to polyvinylidene difluoride membranes for 120 min. The blots were then blocked and incubated with rabbit anti-eNOS polyclonal antibody (0.1 *μ*g/mL, Santa Cruz Biotechnology) for 120 min at room temperature. Next, the blots were incubated with horseradish peroxidase-conjugated goat anti-rabbit IgG (0.08 *μ*g/mL, Santa Cruz Biotechnology). The antibody was visualized using an enhanced chemiluminescence method (ECL, Amersham Biosciences). Computer-assisted densitometry (ImageJ) was used to quantify the bands that were captured on radiographic film.

### 2.7. Determination of Glomerular NO Level and eNOS Activity

 NO test kit and NOS activity classifying kit (Nanjing Jiancheng Bioengineering Institute, China) were used to detect NO level as well as eNOS and iNOS activities in the glomerulus. Procedures were performed in accordance with kit instructions.

### 2.8. Determination of Oxidative Stress Biomarkers in Glomerul

Glutathione (GSH), catalase (CAT), and superoxide dismutase (SOD) enzymes, as well as malondialdehyde (MDA) in the glomeruli were determined according to the instructions on the commercial kits (Nanjing Jiancheng Bioengineering Institute, China).

### 2.9. Statistical Analyses

 Table and graphical data are displayed as the mean ± SD. All parameters were evaluated with the two-tailed unpaired Student's  *t*-test or compared by one-way ANOVA when a multiple mean comparison was required. Correlated coefficients were determined using linear regression analysis. A *P* value < 0.05 denoted the presence of a statistically significant difference.

## 3. Results

### 3.1. Changes in Urinary Albumin Excretion

To evaluate the level of albuminuria in different groups, urinary albumin concentration was expressed as urinary albumin to creatinine ratio (UACR). As compared with normal control group, the rats in DN model group showed a marked elevation of UACR (483.7 ± 87.4 versus 155.3 ± 37.8 *μ*g/mg creatinine). Propyl gallate treatment significantly suppressed UACR of DN rats when compared with DN model group (261.9 ± 23.5 versus 483.7 ± 87.4 *μ*g/mg creatinine). These results suggest the propyl gallate's role in albuminuria reduction.

### 3.2. Histology Analysis

**Figure 1:**
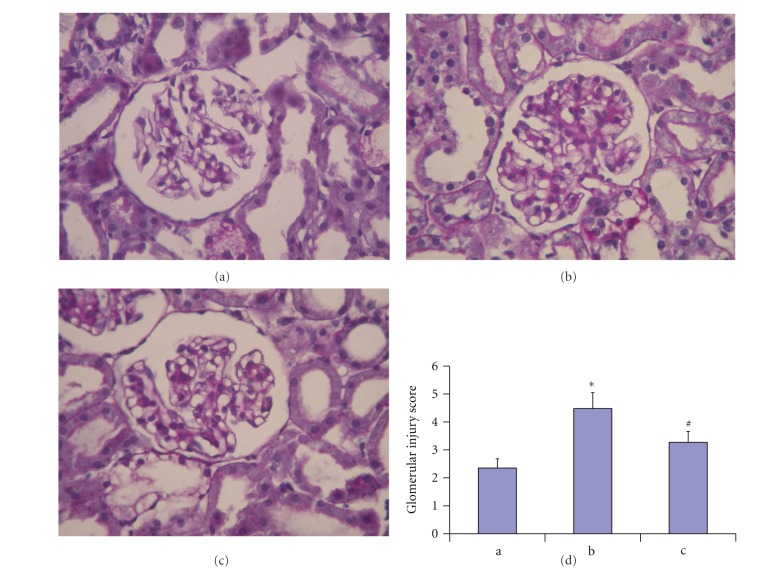
Light microscope appearance of glomeruli for nondiabetic control rat (a), diabetic rat (b), and diabetic rat treated with propyl gallate (c). Number of glomerular capillary, volume of glomeruli, and mesangial area were increased in diabetic rats. However, the increases were diminished in diabetic rat treated with propyl gallate.

 Histological examination of the kidneys shown by PAS staining revealed glomerular hypertrophy and expansion of mesangial area induced by STZ. As shown in Figure 1, Propyl gallate treatment dramatically inhibited glomerular hypertrophy and mesangial expansion induced by STZ. These results suggest the protective role of propyl gallate in DN.

### 3.3. Immunofluorescent Labeling of Glomerular Endothelial Cell

**Figure 2:**
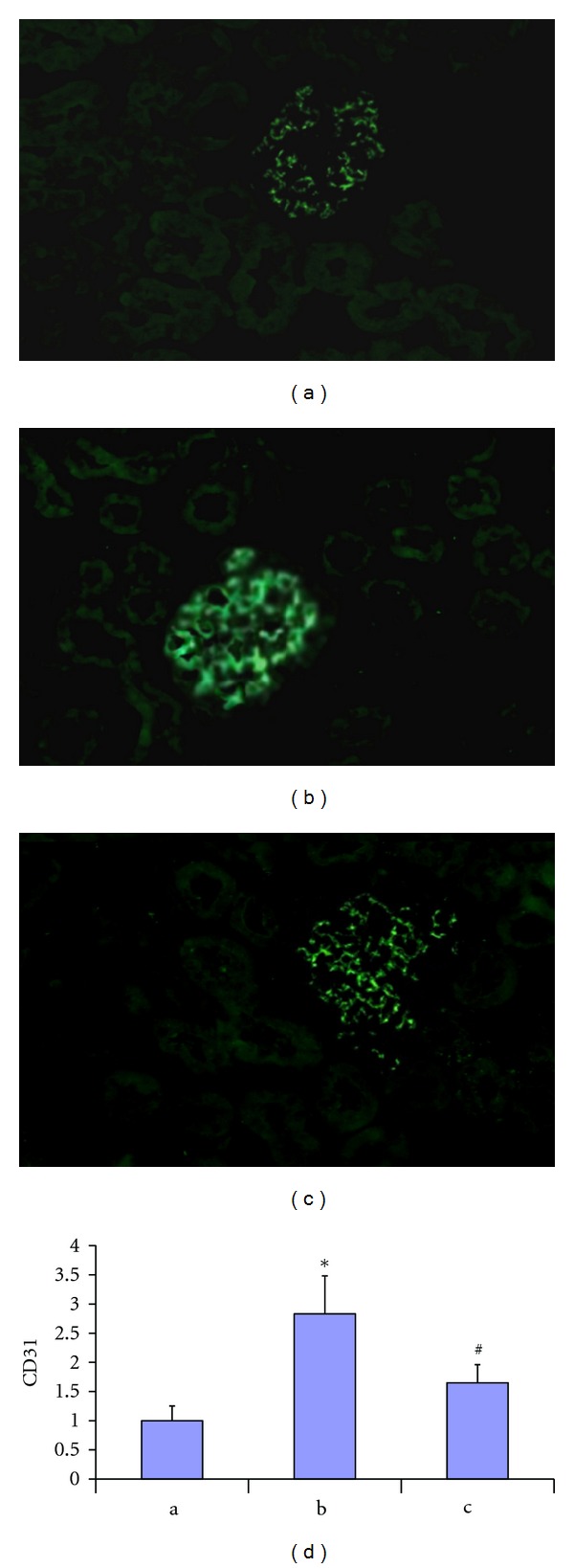
Immunofluorescent staining of CD31 in nondiabetic rat (a), diabetic rat (b), and diabetic rat treated with propyl gallate (5 mg/Kg body wt). The amount of CD31 positive area was significantly increased in diabetic rats. However, the increase was diminished in diabetic rat treated with propyl gallate.

 Glomerular endothelial cells were quantified by CD31 immunofluorescent staining. In nondiabetic rats, CD31 positive staining was detected in glomerular capillaries (Figure 2(a)). The amount of the CD31+ glomerular endothelial area was significantly increased in DN model group (Figure 2(b)), and the quantity of glomerular endothelial area was partially restored by propyl gallate treatment (Figure 2(c)). The amount of CD31 positive endothelial area correlated with the degree of albuminuria for diabetic rat (*r* = 0.53, *P* < 0.05).

### 3.4. Concomitancy of Endothelial Cell and Podocyte in DN Glomerular Vessels

**Figure 3:**
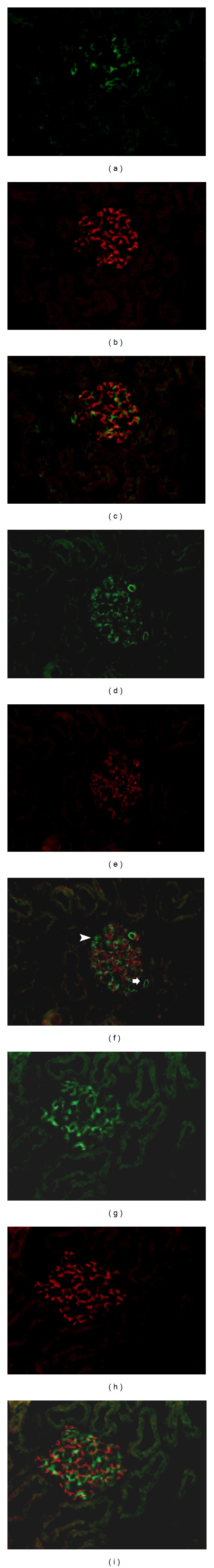
Double immunofluorescent staining of CD31 (green, an endothelial cell marker) and PCX (red, a podocyte marker). ((a) and (b)) Control group, (c) ((a) and (b)) Merged. ((d) and (e)) Diabetic nephropathy group, (f) ((d) and (e)) Merged. ((g) and (h)) Diabetic nephropathy treated with propyl gallate group, (i) ((g) and (h)) Merged. Enhanced CD31 staining was observed in diabetic rat glomeruli. The PCX staining was mostly adjacent to CD31 staining, but there were some CD31 staining areas lacked adjacent PCX staining (arrow and arrowhead) in diabetic nephropathy model group.

 By double labeling of endothelial cell and podocyte, most CD31+ endothelial area was adjacent to PCX+ podocyte area in DN rat glomerulus. The endothelial tubes accompanied by podocyte suggest the mature of glomerular vessels in structure. But there are some areas present nontubulating CD31+ staining, missing adjacent PCX staining. Meanwhile, some CD31 tubulating area also lacked adjacent PCX staining. These two types of glomerular vessels showed the immature of glomerular angiogenesis in structure (Figure 3).

### 3.5. Expression of eNOS mRNA and Protein

**Figure 4:**
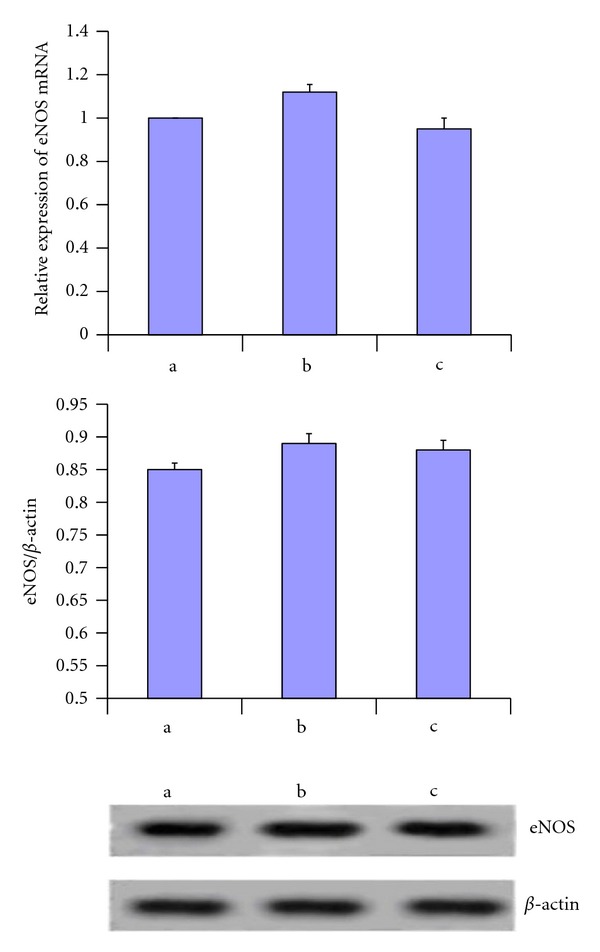
Expression of eNOS mRNA and protein. a: expression of eNOS mRNA detected by real-time PCR. Real-time RT-PCR demonstrated that mRNA levels of eNOS remained at same level in model group rats as compared with normal control rats. Propyl gallate treatment did not alter eNOS mRNA expression. b and c: western blot analysis also showed that the protein levels of eNOS were not significantly changed in model group and propyl gallate treatment group.

The mRNA and protein expression of eNOS was assessed by real-time RT-PCR and western blot analysis. Real-time RT-PCR demonstrated that mRNA levels of eNOS remained at same level in model group rats as compared with normal control rats. Propyl gallate treatment did not alter eNOS mRNA expression. Western blot analysis also showed that the protein levels of eNOS were not significantly changed in model group and propyl gallate treatment group (Figure 4).

### 3.6. Glomerular NO Level and eNOS Activity

Glomerular NO synthesis was evaluated in glomerular homogenate.  Table 1 shows that the glomerular NO analysis demonstrated remarkable reduction in glomerular NO synthesis in diabetic rats compared with that in nondiabetic rats (control group). Propyl gallate significantly prevented the decline. Using an NOS activity classifying test kit, a significant decline in eNOS activity was observed in DN glomeruli compared with those in rats from the control group. Propyl gallate treatment partially restored glomerular eNOS activity, but not to a normal range.

### 3.7. Glomerular Oxidative Stress

Oxidative stress was confirmed by the glomerular homogenate measurements of MDA, SOD, CAT, and GSH-Px. As shown in Table 2, the DN group exhibited decreased SOD, CAT, and GSH-Px levels and increased MDA levels compared with the normal control group. Propyl gallate improved glomerular oxidative stress, restored SOD, CAT, and GSH-Px levels, and decreased MDA levels. By correlation analysis, eNOS activity was inversely correlated with oxidative parameter MDA (*r* = 0.57, *P* < 0.05).

## 4. Discussion

Glomerular hypertrophy has been well characterized in the early STZ-induced diabetes. The morphological phenomena observed in diabetic glomerular hypertrophy include capillary elongation and increased glomerular capillary number, suggesting the involvement of angiogenic process in analogy with diabetic retinopathy [3]. The formation of new blood vessels is composed of several steps: (1) the degradation of vascular basement membrane matrix by protease, (2) migration and proliferation of endothelial cells, (3) endothelial tube formation, (4) recruitment and attachment of mesenchymal cells to the tube, and (5) maturation of blood vessels [11]. The attachment of mesenchymal cells to endothelial tube and differentiation to “pericyte” is a key step to form nonleaky mature blood vessel [12]. It is well known that renal glomeruli are composed of three types of resident cell: endothelial, mesangial, and epithelial cells (podocytes). Mature glomerular capillary vessel wall is constituted by endothelial and epithelial cells. In the setting of diabetic nephropathy, increased endothelial cell proliferation and angiogenesis may facilitate structurally defective vessels. To date, the structure characteristics of newly formed glomerular capillary vessels and its role in the process of diabetic nephropathy advance are not studied considerably.

In this study, characteristic changes in early diabetic nephropathy such as increased urinary albumin excretion and glomerular hypertrophy were observed in diabetic rats. We found significantly increased CD31-positive glomerular endothelial cells in diabetic group. We also found that the levels of albuminuria were correlated with CD31 positive endothelial area. These results indicated the glomerular endothelial proliferation may contribute to glomerular filtration membrane damage in the setting of diabetic nephropathy. Considering the role of albuminuria in diabetic nephropathy progression, it is possible that glomerular endothelial proliferation is a promoting factor in the process of diabetic nephropathy progression.

Although the potential role of endothelial cell proliferation in the pathogenesis of diabetic nephropathy was hinted in this and other studies, how these glomerular endothelial cell proliferation contribute to albuminuria and diabetic nephropathy progression is not investigated extensively. We speculate that rampant endothelial cell proliferation results in excessive angiogenesis and immature vessels in glomeruli in early stage of diabetic nephropathy. Cancer angiogenesis is characterized by morphologically abnormal, immature, dilated, and leaky vessels [13]. These immature vessels lack proper periendothelial coverage by pericytes or smooth muscle cells (SMC). This structure immature results in function immature in these neoangiogenic vessels (such as leaky property). Therefore, analyzing changes in vascular structure in glomerular newly formed vessels might be informative on the potential role of neoangiogenic vessels in the pathogenesis of diabetic nephropathy. Our results showed that there was a kind of endothelial tube formation in diabetic rat glomeruli. Unlike the mature glomerular capillary tube, these immature endothelial tubes lack adjacent podocyte coating. Although we have no direct evidence to confirm the defective capillary tube is derived from newly formed vessels, the imbalance of proliferative endothelial cell and nonproliferative podocyte provide us a postulation for these podocyte-lacking capillary tube formations in diabetic milieu. In diabetic retinopathy, pericyte loss in newly formed vasculus was considered to be the major cause of increased vascular permeability [14]. In our study, the significant correlation between vascular area and level of albuminuria indicated the immature newly formed vessels may also contribute to glomerular vessel leakage. In addition, in synovium of rheumatoid arthritis, active neovascularization formed immature vessels lacking periendothelial coverage, and the progression of the disease was also associated with density of immature vessels [15]. The linkage of these information provide us with a version of speculation to explain how glomerular angiogenesis plays a role in diabetic nephropathy progression.

The mechanism by which glomerular endothelial cell proliferate has received increasing attention. VEGF and NO are two important factors involved in endothelial cell survival and behavior, and one factor cooperates with or opposes the other to maintain endothelial cell homeostasis in the glomerulus. Uncoupling of these two factors is considered a key contributor to the pathogenesis of DN [16]. In many chronic kidney diseases, VEGF levels are low and associated with impaired angiogenesis with capillary loss [17]. Conversely, in most animal models of DN, VEGF levels are elevated [18], suggesting a left-leaning VEGF-NO axis. In our study, we found reduced NO production in diabetic glomerulus. This dual-direction regulation of the VEGF-NO axis may contribute to endothelial cell proliferation.

In diabetes, NO is mainly produced from eNOS in the glomerulus; thus, the factors involved in eNOS deficiency were intensively explored. A number of studies have examined renal NOS expression in diabetes. In the early phase of diabetes, eNOS expression was found elevated [19–21] or unchanged [22–24]. The discrepancy may be explained by the duration of the diabetes, metabolic control, or the presence or absence of insulin treatment. Eight weeks after induction of diabetes mellitus in the rats, we found that the eNOS expression was unchanged. However, the stabilization of eNOS expression did not mean stable NO production in the glomerulus. We found impaired eNOS activity and reduced NO production. Our results imply that the major cause of NO deficiency in diabetic glomerulus is the dysfunction of eNOS; examination on the eNOS expression level was not too much significant. We supposed that environmental factors may influence eNOS activity. Thus, In our study, we examined intraglomerular oxidative stress and found that eNOS activity was negatively correlated with oxidative parameter MDA. These data indicate that oxidative stress may be an important contributor to eNOS activity impairment. Aside from these environmental factors, some studies showed that posttranslational modification may also affect eNOS activity [25].

Propyl gallate is a strong antioxidative agent in many studies [26]. In our present study, we test its effect on endothelial cell proliferation in diabetic nephropathy. We observed a significant inhibitory effect on endothelial cell proliferation in rat diabetic nephropathy. We also found that although propyl gallate did not alter the eNOS expression, it restored the impaired activity of eNOS, and this role may be mediated by its anti-oxidative effect. These finding supported the possibility that propyl gallate, through recovery VEGF-NO axis, inhibit glomerular angiogenic response, block vessel wall permeability, and plays a therapeutic role in diabetic nephropathy.

In conclusion, the present study gives us insight into glomerular endothelial cell proliferation and angiogenesis in the early stage of diabetic nephropathy. Because of the active endothelial cell proliferation, glomerular newly formed capillary vessel may be a defective immature vessel. The defect in structure leads to increased permeability in these vessels and may result in albuminuria. Propyl gallate has a therapeutic effect on diabetic glomerular endothelial proliferation and neovascularization and may be a candidate therapeutic agent in diabetic nephropathy.

## Figures and Tables

**Table 1:** NO production and eNOS activity in different groups (means ± SD). Data show reduced NO production and decreased eNOS activity in DN glomeruli, and propyl gallate restored eNOS activity and NO production.

	control group	DN group	PG group
NO (*μ*mol/L)	127.36 ± 45.85	55.81 ± 16.82^∗^	95.38 ± 22.16^#^
eNOS activity (U/mL)	58.42 ± 11.17	25.74 ± 7.37^∗^	39.31 ± 8.49^#^

^∗^Significantly different compared to control group (*P* < 0.05). ^#^Significantly different compared to DN group (*P* < 0.05).

**Table 2:** Oxidative stress parameters in different groups (means ± SD). Data show increased oxidative stress in DN glomeruli, and propyl gallate treatment improve the intra-glomerular oxidative state.

group	MDA (nmol/mgpro)	Cu Zn—SOD (NU/mgpro)	CAT (U/mgpro)	GSH—Px (U/mgpro)
Control group	3.57 ± 0.14	14.38 ± 3.57	7.43 ± 1.95	11.35 ± 3.37
DN group	9.48 ± 1.33^∗^	7.84 ± 2.58^∗^	3.74 ± 0.57^∗^	7.73 ± 1.63^∗^
PG group	6.38 ± 1.81^#^	10.15 ± 1.41^#^	5.33 ± 0.82^#^	10.51 ± 2.73^#^

^∗^Significantly different compared to control group (*P* < 0.05). ^#^Significantly different compared to DN group (*P* < 0.05).
